# Prophylactic Intra-Uterine β-Cyclodextrin Administration during Intra-Uterine *Ureaplasma parvum* Infection Partly Prevents Liver Inflammation without Interfering with the Enterohepatic Circulation of the Fetal Sheep

**DOI:** 10.3390/nu12051312

**Published:** 2020-05-05

**Authors:** Cathelijne Heymans, Lara R. Heij, Kaatje Lenaerts, Marcel den Dulk, Mhamed Hadfoune, Chantal van Heugten, Owen B. Spiller, Michael L. Beeton, Sarah J. Stock, Alan H. Jobe, Matthew S. Payne, Matthew W. Kemp, Boris W. Kramer, Jogchum Plat, Wim G. van Gemert, Tim G.A.M. Wolfs

**Affiliations:** 1 Department of Surgery, NUTRIM School of Nutrition and Translational Research in Metabolism, Maastricht University, 6200 MD Maastricht, The Netherlands; c.heymans@maastrichtuniversity.nl (C.H.); l.heij@maastrichtuniversity.nl (L.R.H.); kaatje.lenaerts@maastrichtuniversity.nl (K.L.); m.hadfoune@maastrichtuniversity.nl (M.H.); chantalvanheugten@hotmail.com (C.v.H.); wim.van.gemert@mumc.nl (W.G.v.G.); 2 Department of Surgery, University Hospital Aachen, 52074 Aachen, Germany; marcel.den.dulk@mumc.nl; 3 Department of Pathology, University Hospital Aachen, 52074 Aachen, Germany; 4 Department of Surgery, Maastricht University Medical Center, 6202 AZ Maastricht, The Netherlands; 5 Division of Infection and Immunity, School of Medicine, Cardiff University, Cardiff CF10 3AT, UK; spillerb@cardiff.ac.uk; 6 Cardiff School of Sport and Health Sciences, Cardiff Metropolitan University, Cardiff CF5 2YB, UK; mbeeton@cardiffmet.ac.uk; 7 Usher Institute, University of Edinburgh, Edinburgh EH16 4TJ, UK; sarah.stock@ed.ac.uk; 8 Division of Obstetrics and Gynecology, The University of Western Australia, Crawley WA 6009, Australia; alan.jobe@cchmc.org (A.H.J.); matthew.payne@uwa.edu.au (M.S.P.); matthew.kemp@uwa.edu.au (M.W.K.); 9 Division of Neonatology/Pulmonary Biology, The Perinatal Institute, Cincinnati Children’s Hospital Medical Center, University of Cincinnati, Cincinnati, OH 45229, USA; 10 School of Veterinary and Life Sciences, Murdoch University, Perth WA 6150, Australia; 11 Department of Pediatrics, School for Oncology and Developmental Biology (GROW), Maastricht University, 6200 MD Maastricht, The Netherlands; b.kramer@maastrichtuniversity.nl; 12 Neonatology, Department of Pediatrics, Maastricht University Medical Center, 6202 AZ Maastricht, The Netherlands; 13 Department of Nutrition and Movement Sciences, NUTRIM School of Nutrition and Translational Research in Metabolism, Maastricht University, 6200 MD Maastricht, The Netherlands; j.plat@maastrichtuniversity.nl; 14 Department of Biomedical Engineering (BMT), School for Cardiovascular Diseases (CARIM), Maastricht University, 6200 MD Maastricht, The Netherlands

**Keywords:** *Ureaplasma parvum*, intra-uterine infection, chorioamnionitis, preterm birth, plant sterols, liver, enterohepatic circulation, sheep

## Abstract

Chorioamnionitis can lead to inflammation and injury of the liver and gut, thereby predisposing patients to adverse outcomes such as necrotizing enterocolitis (NEC). In addition, intestinal bile acids (BAs) accumulation is causally linked to NEC development. Plant sterols are a promising intervention to prevent NEC development, considering their anti-inflammatory properties in the liver. Therefore, we investigated whether an intra-amniotic (IA) *Ureaplasma parvum* (UP) infection affected the liver and enterohepatic circulation (EHC) and evaluated whether an IA administered plant sterol mixture dissolved in β-cyclodextrin exerted prophylactic effects. An ovine chorioamnionitis model was used in which liver inflammation and the EHC were assessed following IA UP exposure in the presence or absence of IA prophylactic plant sterols (a mixture of β-sitosterol and campesterol dissolved in β-cyclodextrin (carrier)) or carrier alone. IA UP exposure caused an inflammatory reaction in the liver, histologically seen as clustered and conflated hepatic erythropoiesis in the parenchyma, which was partially prevented by IA administration of sterol + β-cyclodextrin, or β-cyclodextrin alone. In addition, IA administration of β-cyclodextrin prior to UP caused changes in the expression of several hepatic BAs transporters, without causing alterations in other aspects of the EHC. Thereby, the addition of plant sterols to the carrier β-cyclodextrin did not have additional effects.

## 1. Introduction

Preterm birth, birth before 37 weeks of gestation, is the leading cause of morbidity and mortality among infants worldwide [1]. An important cause of preterm birth is chorioamnionitis, an inflammatory cell infiltration of the fetal membranes, which is defined as an independent risk factor for the development of necrotizing enterocolitis (NEC) [2,3,4,5]. The most common organism isolated from the amniotic fluid of pregnant women with chorioamnionitis is *Ureaplasma parvum* (UP), a commensal of the urogenital tract of humans [6,7,8]. UP colonization in preterm infants has been associated with an increased incidence of NEC [2,3,4,5]. 

A strong interaction exists between the intestine and liver, wherein a crucial role is played by the enterohepatic circulation (EHC) of bile acids (BAs). There is increasing attention towards the role of BAs as critical regulators of intestinal epithelial function [9]. Within this context, their contribution to NEC pathophysiology has recently been studied. In the week preceding NEC manifestation, fecal unconjugated BA levels were found to be higher in preterm infants, eventually developing NEC compared with gestation-matched controls [10]. More precisely, the intraluminal accumulation of conjugated BAs resulted in intestinal epithelial damage, similar to the histopathological findings in NEC [11]. These high concentrations of intraluminal BAs might be the result of increased BA synthesis, as was shown in a rat NEC model [12]. In addition, the increased expression of the apical sodium-dependent bile acid transporter (*ASBT*) in the terminal ileum, a protein involved in the uptake of conjugated BAs into the enterocytes, was reported in preterm infants with NEC and in an experimental NEC model with rodents which correlated with the location of intestinal damage, suggesting increased BAs uptake by enterocytes [13,14]. Consequently, high concentrations of intraluminal BAs resulted in their accumulation within enterocytes, with concomitant enterocyte damage [10]. Moreover, a decreased expression of the ileal bile acid-binding protein (*IBABP*) was seen in the terminal ileum of rats in an experimental NEC model, suggesting insufficient transport from the apical to the basolateral side of enterocytes with consequent BAs accumulation within the enterocytes [14]. The important role of the liver and the gut-liver axis in NEC pathogenesis is further underlined by the presence of increased hepatic inflammation in neonatal rats with NEC, which correlated with the progression of intestinal damage during disease development [15]. 

Interestingly, in a recent study, chorioamnionitis induced by six days of intra-uterine UP exposure caused a reduced amount of conjugated BAs in the enterocytes of the terminal ileum of fetal sheep [16]. In addition, it was shown that fetuses exposed to endotoxin-induced chorioamnionitis develop hepatic inflammation and a disturbed lipid metabolism *in utero* [17]. These findings prompted us to investigate the liver and EHC alterations in a model of UP-induced chorioamnionitis in fetal sheep. 

From a treatment perspective, plant sterols, which are dietary constituents present in vegetable oils, nuts, grains, and fruit [18], seem promising due to their immune-modulatory properties [19,20,21,22,23,24,25,26,27]. Moreover, they have even shown to be anti-inflammatory in the liver of mice that developed non-alcoholic fatty liver disease (NASH) [28]. We previously showed that fetal gut inflammation and mucosal damage following IA UP exposure were prevented by prophylactic plant sterol supplementation in the amniotic fluid [16]. Therefore, the second aim of this study was to investigate the effects of prophylactic IA administration of a plant sterol mixture dissolved in β-cyclodextrin as carriers on liver inflammation and potential EHC alterations.

## 2. Materials and Methods 

### 2.1. Animal Model and Experimental Procedures 

**Figure 1:**
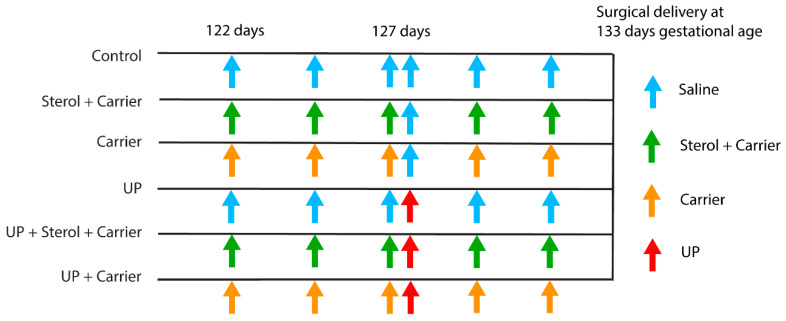
Experimental design. Animals were randomly assigned to six different study groups; control: *n* = 6, plant sterols in carrier: *n* = 6, carrier alone: *n* = 6, *Ureaplasma parvum* (UP): *n* = 7, UP + plant sterols in carrier: *n* = 7, UP + Carrier alone: *n* = 7. Plant sterols dissolved in the carrier (β-cyclodextrin) were administered by an intra-amniotic (IA) injection at 122d of gestational age (GA), before the onset of chorioamnionitis and were repeated every two days until 131d GA. The animals were prematurely delivered at 133d GA. Two groups received the carrier alone (without added plant sterols) IA to assess the effects of the carrier separately from the plant sterols. *Ureaplasma parvum* serovar 3 (10^7^ color-changing units [CCU]) was given by IA injection at 127d GA to induce chorioamnionitis. The control group received saline on all administration time points.

The animal experiments were approved by the Animal Ethics Committee of the University of Western Australia (Perth, Australia; ethical approval code: RA/3/100/1378). The animal model and experimental procedures were previously described [16]. In short, fifty ewes with singleton fetuses were randomly assigned to the six different study groups (Figure 1). After drop-outs, the group size for data analyses was six to seven animals per group. One group of the date-mated pregnant ewes received an IA ultrasound-guided injection of *Ureaplasma parvum* serovar 3 (strain HPA5, 10^7^ color-changing units [CCU]), six days prior to delivery. To evaluate the preventive effect of plant sterols, the amniotic fluid in two experimental groups was enriched with a mixture of β-sitosterol (70%) and campesterol (30%) (total of 0.6 mg/mL), dissolved in a carrier, 18% 2-hydroxypropyl-β-cyclodextrin (H107, Sigma Aldrich, St. Louis, MO, USA) eleven days prior to delivery in combination with saline, or UP six days prior to delivery. To assess the carrier effect separately from the plant sterols, in two groups, the carrier was given alone with saline or carrier in combination with UP. A group receiving IA injections of saline (eleven or six days prior to delivery) served as control (Figure 1).

Fetuses were delivered preterm by cesarean section at 133 days of gestational age (term ewe gestation = 150 days), the equivalent of 33–34 weeks of human gestation. After cesarean section, fetuses were euthanized directly with intravenous pentobarbital (100 mg/kg, Valabarb, Jurox, Rutherford, NSW, Australia). During necropsy, blood, liver, and terminal ileum samples were sampled. 

### 2.2. Qualitative Analysis of Liver Histology 

H&E slides were scored on a zero to four scale for degree of hepatic sinusoidal dilatation, shape and size of central veins, and number and location of extramedullary hematopoietic clusters by an independent pathologist blinded to the experimental set-up. Zero represented no sinusoidal dilatation, no divergent shape or size of central veins, and no inflammation. Four represented the other end of the scale, namely pronounced sinusoidal dilatation throughout the parenchyma, large central veins with venous stowing throughout the parenchyma, and a severely increased number of clusters of hematopoiesis.

### 2.3. Total Bile Acid Assay

Total bile acids (tBAs) in plasma and liver homogenate were measured by an enzymatic cycling method using the Total Bile Acids Assay kit, according to the manufacture protocol (Diazyme Laboratories, Poway, CA, USA). tBAs in liver homogenate were corrected for protein content.

### 2.4. RNA Extraction and Real-Time PCR 

RNA was isolated from snap-frozen liver and ileum tissue using TRI reagent (Thermo Fisher Scientific, Waltham, MA, USA)/chloroform extraction. The RNA was reverse transcribed into cDNA using a sensifast cDNA Synthesis kit (Bioline, London, UK). Quantitative real-time PCR (qPCR) was executed with the selected primer in Sensimix SYBR & Fluorescein Kit (Bioline, London, UK). The qPCR reactions were performed with the use of the LightCycler 480 Instrument (Roche Applied Science, Basel, Switzerland) for 45 cycles. The gene expression levels of cholesterol 7 alpha-hydroxylase (*CYP7A1*), Cytochrome P450 Family 27 Subfamily A Member 1 (*CYP27A1*), Na+-taurocholate cotransporting polypeptide (*NTCP*), bile salt export pump (*BSEP*), apical sodium–dependent bile acid transporter (*ASBT*), fibroblast growth factor 19 (*FGF19*), ileal bile acid-binding protein (*IBABP*) and organic solute transporter alpha-beta (*OSTα-β*) were determined to assess changes in the EHC. LinRegPCR software (version 2016.0, Heart Failure Research Center, Academic Medical Center, Amsterdam, the Netherlands) was used to calculate the expression levels. As a normalization factor, the geometric mean of the expression levels of three reference genes (ribosomal protein S15 (*RPS15*), glyceraldehyde 3-phosphate dehydrogenase (*GAPDH*) and peptidylprolyl isomerase A (*PPIA*)) was calculated. The data were expressed as a fold increase over the control value, arbitrary unit (AU). An overview of the used primer sequences is shown in Table 1.

### 2.5. Data Analysis

Statistical analyses were performed using GraphPad Prism (version 6.01, GraphPad Software Inc., La Jolla, CA, USA). Data are presented as median with interquartile range (IQR). Differences in the qualitative analysis of liver histology using scorings were assessed using the Kruskal–Wallis test followed by Dunn’s post hoc test. A square root transformation was applied to the other data to obtain a normal distribution. A comparison between different experimental groups was performed using the two-way ANOVA followed by Tukey’s or Sidak’s multiple comparisons test. Differences were considered statistically significant at *p* < 0.05.

## 3. Results

### 3.1. Sterol + Carrier, as Well as Carrier Alone, Partly Decrease Hepatic Inflammation Due to UP-Induced Chorioamnionitis

**Figure 2:**
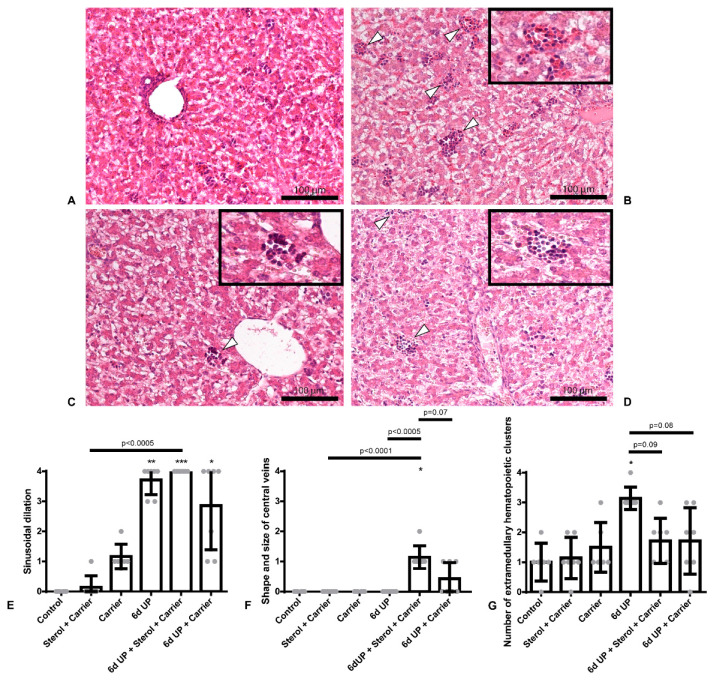
H&E slides scored on a zero to four scale for degree of sinusoidal dilatation, shape and size of central veins, and number and location of extramedullary hematopoietic clusters by an independent pathologist. Representative images of the control (**A**), UP (**B**), UP + sterols dissolved in carrier (**C**) and UP + carrier alone (**D**). (**E**): Increased sinusoidal dilation in animals exposed to UP, UP + sterols dissolved in the carrier, and UP + carrier alone. * *p* < 0.01, ** *p* < 001, *** *p* < 0.0005 compared to control. (**F**): The UP + sterol dissolved in the carrier group displayed enlarged central veins, without shape divergence. * *p* = 0.0002 compared to control. (**G**): Increased number of extramedullary hematopoietic clusters in the animals exposed to UP. Treatment with prophylactic IA sterols dissolved in the carrier or carrier alone prior to UP exposure tended to decrease the number of extramedullary hematopoietic clusters. Specifically, the extramedullary hematopoiesis in all the animals exposed to UP is interpreted as clustered and conflated hepatic erythropoiesis in the parenchyma, which are indicated by white triangles. * *p* = 0.001 compared to control.

Histologically, no signs of sinusoidal dilatation, divergent shape, or size of central veins or inflammation were observed in the control group and animals exposed to the sterol + carrier or carrier alone (Figure 2E–G). All animals exposed to UP (all groups) displayed pronounced sinusoidal dilation throughout the entire liver parenchyma (all *p* < 0.01; Figure 2E) without dilatation of the central veins. Administration of sterol + carrier or carrier alone prior to UP exposure did not decrease sinusoidal dilation. However, the animals treated with sterol + carrier prior to UP displayed enlarged central veins compared to control, sterol + carrier, UP, and UP + carrier (all *p* < 0.10; Figure 2F), without shape divergence. 

A significant increase in the number of erythropoietic clusters was observed in animals exposed to UP (*p* < 0.005; Figure 2G), as the extramedullary hematopoiesis is interpreted as clustered and conflated hepatic erythropoiesis in the parenchyma (Figure 2B–D). The administration of sterol + carrier or carrier alone prior to UP exposure tended to have lower numbers of erythropoietic clusters (both *p* < 0.10; Figure 2G), suggesting that the carrier exerted this anti-inflammatory effect while dissolving sterols in the carrier did not have an additional effect on top of the carrier. 

### 3.2. Administration of the Carrier Alone Prior to UP Exposure Causes Increased BSEP Expression in the Liver

**Figure 3:**
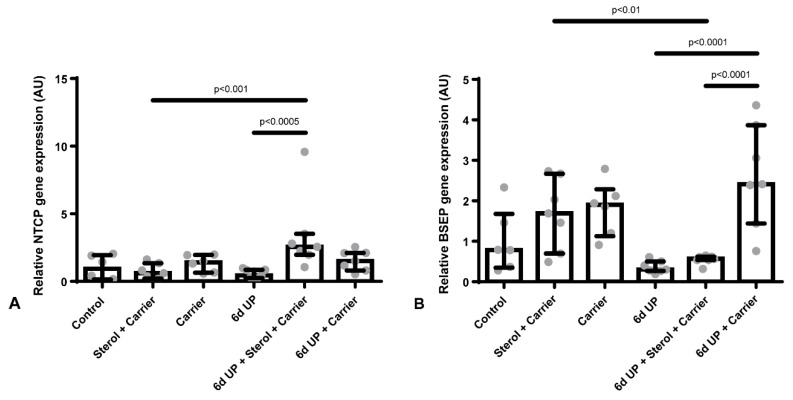
Relative gene expression of Na+-taurocholate cotransporting polypeptide (*NTCP*) (**A**) and bile salt export pump (*BSEP*) (**B**) in AU in the liver. (**A**): Increased *NTCP* gene expression in animals exposed to UP + sterols dissolved in the carrier. (**B**): Increased *BSEP* gene expression in animals exposed to UP + carrier alone.

*NTCP* and *BSEP* mRNA expression levels were not altered in animals exposed to UP alone, compared to control (Figure 3A,B). However, in the UP + carrier group, an increased *BSEP* mRNA expression was observed compared with UP alone or UP + sterol + carrier (*p* < 0.05; Figure 3B).

No differences in the amount of tBAs in plasma or the liver were measured (Supplementary Materials 1: Figure S1). Furthermore, no differences were observed in the mRNA expression of the BAs synthesis markers *CYP7A1* and *CYP27A1* (Supplementary Materials 2: Figure S2). In addition, no differences were observed in the mRNA expression of any of the BAs transporters in the gut (*ASBT*, *OSTα-β*; Supplementary Materials 3: Figure S3), as well as intestinal *FGF19* or *IBABP* (Supplementary Materials 4: Figure S4).

A summary of all the results is given in the synthetic table (Table 2).

## 4. Discussion

Chorioamnionitis induced by IA UP exposure for six days caused an inflammatory reaction in the liver, characterized by increased sinusoidal dilation and an increased number of extramedullary hematopoietic clusters. Specifically, clustering and conflation of hepatic erythropoiesis in the parenchyma were observed in these animals. This phenomenon was also observed in human babies born stillborn, in whom there was an increase in total hematopoiesis and erythropoiesis with clustering of erythropoietic cells, which was strongly associated with chorioamnionitis [29], implicating that antenatal inflammation alters fetal extramedullary hematopoiesis. The observed fetal hepatic cellular response to chorioamnionitis in our study might be the result of the fetal systemic inflammatory response (FIRS; increased circulatory IL-6 levels [16]), or direct exposure to inflammatory mediators through the transport from the gut via the portal vein, or a combination of both. Additionally, the distribution of erythropoiesis was altered in the previously mentioned study in humans, in which erythropoietic activity was often expanded over the whole portal field and the sinusoids in the case of chorioamnionitis [29]. However, in our study, we observed alterations of fetal intrahepatic erythropoiesis in the parenchyma. Whether this difference is species-dependent remains to be elucidated, since no previous data on alterations of fetal intrahepatic erythropoiesis is present in sheep.

In our study, prophylactic IA exposure to carrier or carrier enriched with plant sterols prior to IA UP exposure partly decreased the number of clusters of erythropoiesis in the liver parenchyma. In our study, the carrier β-cyclodextrin exerted similar anti-inflammatory effects in the liver as was previously observed in the gut by plant sterols [16]. The effects in the gut were explained by the plant sterols and the carrier β-cyclodextrin apparently having overlapping working mechanisms [16]. Given the fact that plant sterols dissolved in the carrier, and the carrier alone possessed similar anti-inflammatory effects, our data suggests that the anti-inflammatory effects in the liver might be solely the result of the carrier β-cyclodextrin.

Caution to use plant sterols in the fetal setting of chorioamnionitis might be required, since sterol-treated animals displayed enlarged central veins in combination with pronounced sinusoidal dilatation, implying hepatic congestion [30]. However, not significant in the case of carrier administration alone, these effects were also visible elsewhere, concluding that one should be cautious here as well.

A previous study in the same model showed that chorioamnionitis induced by six days of IA UP exposure caused a reduction of conjugated BAs in the enterocytes of the terminal ileum of fetal sheep in the same model [16]. This might indicate that, as a result of chorioamnionitis, the uptake of BAs from the lumen into the enterocyte is reduced, or the amount of BAs in the lumen is reduced by a potential decreased excretion of BAs from the liver into the intestine, or a reduced BA synthesis. However, in our study, none of the above could explain the reduction of conjugated BAs in the enterocytes of the terminal ileum, since mRNA expression levels of the BA transporter responsible for the uptake of conjugated BAs in the terminal ileum, *ASBT*, were unchanged in animals exposed to UP. Moreover, mRNA expression levels of the pump responsible for the excretion of BAs from the hepatocytes into the bile canaliculi, *BSEP*, remained unaltered in animals exposed to UP alone.

Our findings suggest that the BAs synthesis in the liver of UP-exposed animals remained unchanged. This is an interesting finding since we previously demonstrated in these animals a reduction of conjugated BAs in enterocytes, and reduced fetal circulatory lathosterol concentrations, indicative of a reduced (hepatic) endogenous cholesterol synthesis [16]. Since BAs are synthesized via oxidation of cholesterol [31], we assumed we would detect an altered syntheses of primary BAs as a result of IA UP exposure. However, the liver cholesterol pool was sufficient to such an extent that it did not have a negative impact on the syntheses of primary BAs. It might be possible that the conjugated BAs that were not absorbed in the terminal ileum of the fetal sheep via the *ASBT* were deconjugated in the colon and subsequently passively absorbed. When the deconjugated BAs would not have been passively absorbed in the colon, they would be lost in the feces. In this case, one would have expected increased BAs syntheses to maintain a constant BA pool, suggesting that there was no loss of BAs in our study.

In our study, we could not observe the EHC alterations found in experimental NEC and neonates with NEC. A possible explanation for this discrepancy could be that the EHC alterations contributing to NEC pathology do not have their origin *in utero* but might manifest after birth.

Finally, sterols dissolved in the carrier or carrier alone did not cause any alterations in the EHC. However, in combination with UP, increased mRNA expression levels of *NTCP* and *BSEP* were found. Although exposure to plant sterols dissolved in the carrier as well as the β-cyclodextrin carrier alone prior to UP caused changes in the expression of several hepatic BAs transporters, this did not cause EHC alterations further up in the circulation, e.g., altered BAs synthesis or altered *ASBT* mRNA expression levels, suggesting that these alterations might not be biologically relevant.

Overall, we can conclude from this study that the observed effects are not the result of the addition of plant sterols to the carrier β-cyclodextrin but can be fully assigned to the carrier β-cyclodextrin alone. We have previously found that plant sterols and the carrier β-cyclodextrin protect the fetal gut against chorioamnionitis induced gut inflammation and injury [16]. Based on these earlier findings and the current study, we conclude that β-cyclodextrin is an interesting pharmaceutical target to protect the gut and liver against the negative consequences of perinatal inflammatory stress [32,33]. Importantly, cyclodextrins are highly water-soluble, “ready-made,” and commercially available. Its pharmacokinetics and the optimal moment and route of administration in the fetal or neonatal context warrant further investigation.

In summary, IA UP exposure caused an inflammatory reaction in the liver, which was partially prevented by IA β-cyclodextrin administration. In addition, IA administration of β-cyclodextrin prior to UP caused changes in the expression of several hepatic BAs transporters, without causing alterations in other aspects of the EHC. Thereby, the addition of plant sterols to the carrier β-cyclodextrin did not have additional effects.

## Figures and Tables

**Table 1:** Primer sequences.

Primer	Forward	Reverse
*RPS15*	5′-CGAGATGGTGGGCAGCAT-3′	5′-GCTTGATTTCCACCTGGTTGA-3′
*GAPDH*	5′-GGAAGCTCACTGGCATGGC-3′	5′-CCTGCTTCACCACCTTCTTG-3′
*PPIA*	5′-TTATAAAGGTTCCTGCTTTCACAGAA-3′	5′-ATGGACTTGCCACCAGTACCA-3′
*CYP7A1*	5′-GGGCATCACAAGCAAACACC-3′	5′-GATGATACTGTCTAGCACGGG-3′
*CYP27A1*	5′- CCCAAGAATACCCAGTTTGTGC-3′	5′- GGTGGCAGAAGACTCAGTTCA-3′
*NTCP*	5′-TCCTCAAATCCAAACGGCCA-3′	5′-GTTTGGATCGTCCATTGAGGC-3′
*BSEP*	5′-ACTCAGTAATTCTTCGCAGTGTG-3′	5′-ATCGAAACAATCGAAAGAAGCCA-3′
*ASBT*	5′-CATGGACCTGAGCGTCAGCAT-3′	5′-CACGGAGACGGGAACAACAA-3′
*FGF19*	5′-TTGATGGAGATCAGGGCGGT-3′	5′-CGGATCTCCTCCTCGAAAGC-3′
*IBABP*	5′-ACAAGAAGTTCAAGGTCACCG-3′	5′-TGATACGGCTTTATGGCCCC-3′
*OSTα*	5′-ATCCCAGGTACACGGCAGAT-3′	5′-ATTGAGGCCAGGACAAGCAA-3′
*OSTβ*	5′-CCGAGTAGAGGATGCAACTCC-3′	5′-TTTGTTTTTCCGGTGGCAGC-3′

**Table 2:** Synthetic table.

	Control	Sterol + Carrier	Carrier	UP	UP + Sterol + Carrier	UP + Carrier
Hepatic sinusoidal dilation	nc	nc	nc	↑	↑ (Control & Sterol + Carrier	↑
Shape and size of central veins	nc	nc	nc	nc	↑ (Control, Sterol + Carrier, UP & UP + Carrier)	nc
Number of extramedullary hematopoietic clusters	nc	nc	nc	↑	↓ (UP)	↓ (UP)
*NTCP*	nc	nc	nc	nc	↑ (Sterol + Carrier & UP)	nc
*BSEP*	nc	nc	nc	nc	nc	↑ (Sterol + Carrier, UP & UP + Sterol + Carrier)
tBAs plasma	nc	nc	nc	nc	nc	nc
tBAs liver	nc	nc	nc	nc	nc	nc
*CYP7A1*	nc	nc	nc	nc	nc	nc
*CYP27A1*	nc	nc	nc	nc	nc	nc
*ASBT*	nc	nc	nc	nc	nc	nc
*OSTα-β*	nc	nc	nc	nc	nc	nc
*FGF19*	nc	nc	nc	nc	nc	nc
*IBABP*	nc	nc	nc	nc	nc	nc

nc: Not changed. ↑ or ↓: Increased or decreased compared to control. ↑ or ↓ (group): Increased or decreased compared to the mentioned group(s).

## Supplementary Materials

The following are available online at https://www.mdpi.com/2072-6643/12/5/1312/s1; Figure S1: tBAs concentrations in plasma and liver, Figure S2: Relative gene expression of *CYP7A1* and *CYP27A1*, Figure S3: Relative gene expression of *ASBT*, *OSTα* and *OSTβ*, Figure S4: Relative gene expression of *FGF19* and *IBABP*.

## Abbreviations

*ASBT*	Apical sodium–dependent bile acid transporter
AU	Arbitrary unit
BAs	Bile acids
*BSEP*	Bile salt export pump
CCU	Color-changing units
*CYP7A1*	Cholesterol 7 alpha-hydroxylase
*CYP27A1*	Cytochrome P450 Family 27 Subfamily A Member 1
EHC	Enterohepatic circulation
*FGF19*	Fibroblast growth factor 19
*GAPDH*	Glyceraldehyde 3-phosphate dehydrogenase
IA	Intra-amniotic
*IBABP*	Ileal bile acid-binding protein
IQR	Interquartile range
NEC	Necrotizing enterocolitis
*NTCP*	Na+-taurocholate cotransporting polypeptide
*OSTα*	Organic solute transporter alpha
*OSTβ*	Organic solute transporter bèta
*PPIA*	Peptidylprolyl isomerase A
*RPS15*	Ribosomal protein S15
tBAs	Total bile acids
UP	*Ureaplasma parvum*
qPCR	Quantitative real-time PCR
