# Supplementary material for: Prophylactic Intra-Uterine β-Cyclodextrin Administration during Intra-Uterine Ureaplasma parvum Infection Partly Prevents Liver Inflammation without Interfering with the Enterohepatic Circulation of the Fetal Sheep
Source: Nutrients. 2020 May 5;12(5):1312. doi: 10.3390/nu12051312 (PMC7284867; doi:10.3390/nu12051312)
Supplement: Supplementary file 1 [file nutrients-12-01312-s001.pdf]

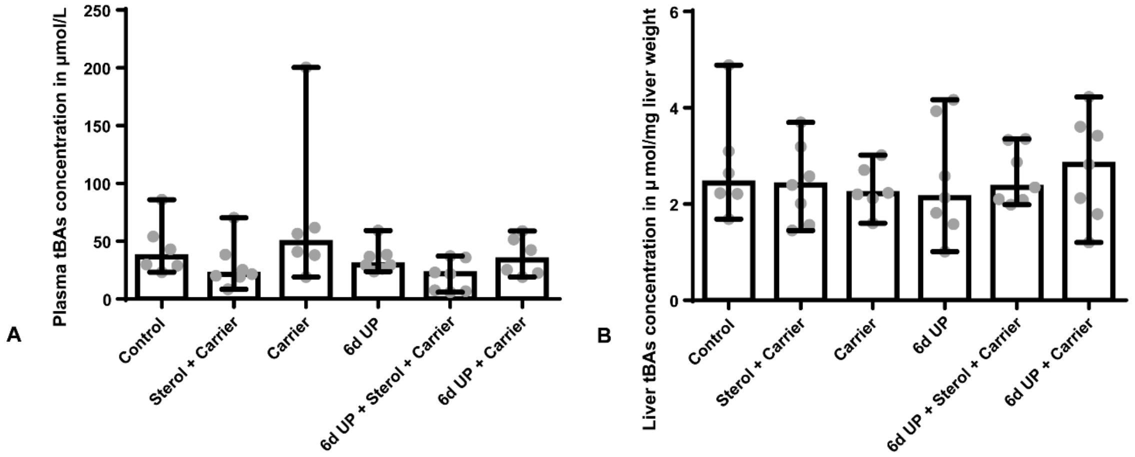

Figure S1. tBAs concentrations in plasma (**A**) in µmol/L and liver (**B**) in µmol/mg liver weight. No differences in the amount of tBAs in plasma or the liver were measured.

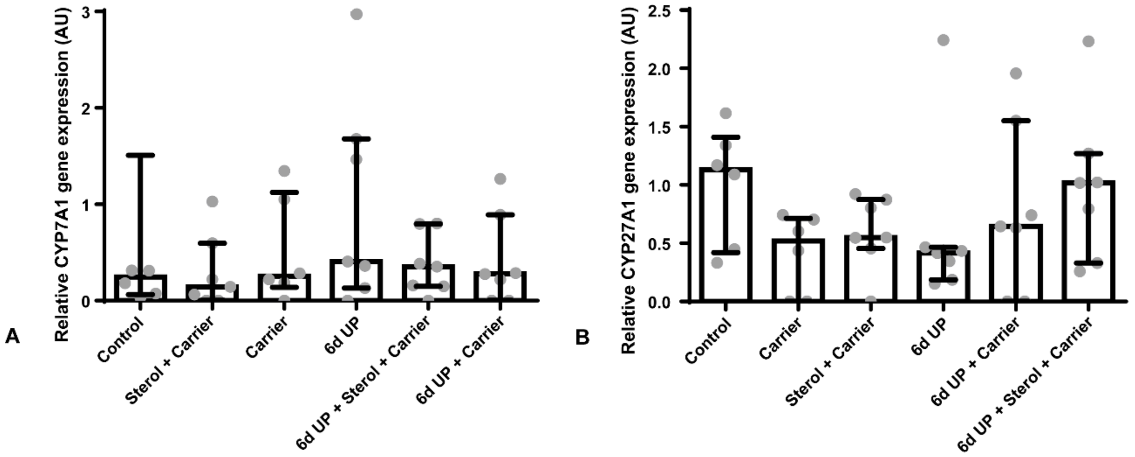

Figure S2. Relative gene expression of CYP7A1 (**A**) and CYP27A1 (**B**) in AU in the liver. No differences were observed in the mRNA expression of the BAs synthesis markers CYP7A1 and CYP27A1.

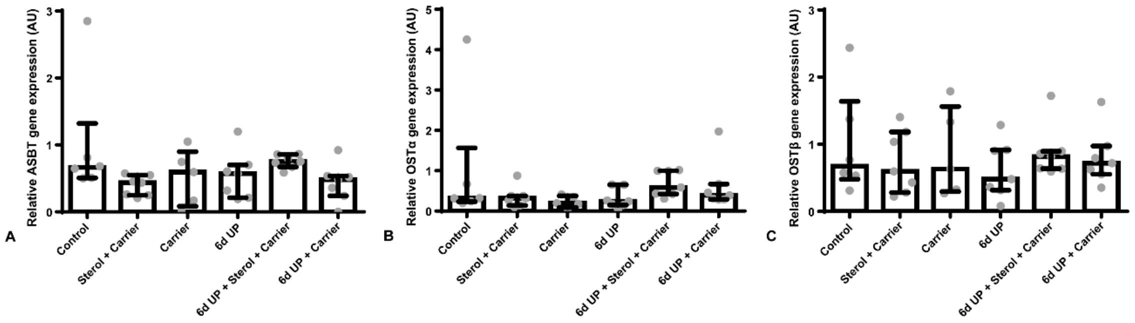

Figure S3. Relative gene expression of ASBT (**A**), OSTα (**B**) and OSTβ (**C**) in AU. No differences were observed in the mRNA expression of any of these BAs transporters in the gut.

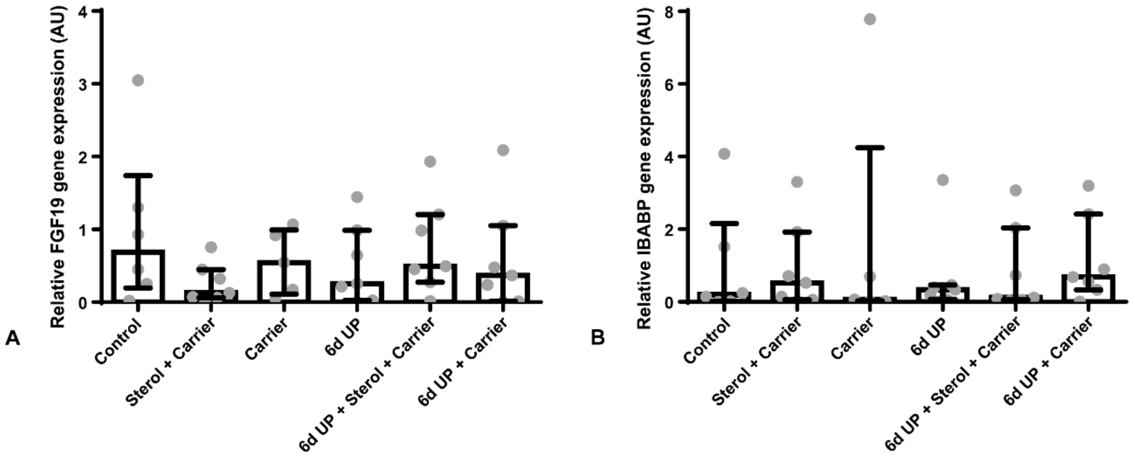

Figure S4. Relative gene expression of FGF19 (**A**) and IBABP (**B**) in AU. No differences were observed in the mRNA expression of intestinal FGF19 or IBABP.
